# 5,6-diiodo-1H-benzotriazole: new TBBt analogue that minutely affects mitochondrial activity

**DOI:** 10.1038/s41598-021-03136-8

**Published:** 2021-12-08

**Authors:** Daniel Paprocki, Maria Winiewska-Szajewska, Elżbieta Speina, Róża Kucharczyk, Jarosław Poznański

**Affiliations:** 1 Institute of Biochemistry and Biophysics, Polish Academy of Sciences, Pawińskiego 5a, 02-106 Warsaw, Poland; 2 Division of Biophysics, Institute of Experimental Physics, University of Warsaw, Pasteura 5, 02-089 Warsaw, Poland

**Keywords:** Biochemistry, Biophysics, Cancer

## Abstract

4,5,6,7-Tetrabromo-1*H*-benzotriazole is widely used as the reference ATP-competitive inhibitor of protein kinase CK2. Herein, we study its new analogs: 5,6-diiodo- and 5,6-diiodo-4,7-dibromo-1*H*-benzotriazole. We used biophysical (MST, ITC) and biochemical (enzymatic assay) methods to describe the interactions of halogenated benzotriazoles with the catalytic subunit of human protein kinase CK2 (hCK2α). To trace the biological activity, we measured their cytotoxicity against four reference cancer cell lines and the effect on the mitochondrial inner membrane potential. The results obtained lead to the conclusion that iodinated compounds are an attractive alternative to brominated ones. One of them retains the cytotoxicity against selected cancer cell lines of the reference TBBt with a smaller side effect on mitochondrial activity. Both iodinated compounds are candidate leaders in the further development of CK2 inhibitors.

## Introduction

Protein kinase CK2 is highly pleiotropic and constitutively active serine/threonine kinase, which catalyzes the phosphorylation of numerous protein substrates, which are often related to gene expression or protein synthesis. CK2 is essential to cell viability, apoptosis, proliferation and survival, angiogenesis, DNA-damage repair, the ER-stress response, the regulation of carbohydrate metabolism and development of nervous system. Many CK2 substrates are essential for a pathogen life cycle^1,2^, so its activity is a subject of long term studies^3–5^. Moreover, kinase CK2 is directly related to cancer research, since its protein substrates have been implicated in several human cancers, including breast, lung, colon, prostate, as well as hematologic malignancies^6^. Human CK2 is the holoenzyme consisting of two catalytic subunits (α and/or α’) and two regulatory ones (β). The activity of this enzyme is associated with the catalytic subunit, while the regulatory ones are responsible for substrate recognition and stabilization of the holoenzyme complex^7^.

4,5,6,7-Tetrabtomo-1*H*-benzotriazole (TBBt or **BBBB**) is widely used as the reference ATP-competitive selective inhibitor of protein kinase CK2^8^. It was originally developed as the analogue of 5,6-dichloro-1-(β-D-ribofuranosyl)benzimidazole (DBR), which was found to inhibit mRNA, but not rRNA or tRNA, synthesis in eukaryotic cells^9,10^. TBBt and its analogue 4,5,6,7-tetrabromo-1*H*-benzimidazole (TBBz) were also starting points for synthesis of potent CK2 inhibitors, including compounds further substituted in five-membered ring (e.g. 4,5,6,7-tetrabromo-2-(dimethylamino)benzimidazole^11^) as well as inhibitor-peptide conjugates^12–15^.

We have already identified 5,6-dibormo-1*H*-benzotriazole (**HBBH**) as a ligand strongly binding to hCK2α^16,17^. Its affinity to hCK2α estimated by Differential Scanning Fluorimetry was very close to that displayed by TBBt^16^. The binding thermodynamics studied with MST and ITC proved that **HBBH** binds to hCK2α minutely weaker than TBBt^17^. The difference in the dissociation constant for the strongly binding site is even much smaller than DSF data suggested. We have also shown that **HBBH** is much less hydrophobic and less acidic than TBBt^18^, which may make it less toxic. Herein, we study the properties of two iodinated compounds: 5,6-diiodo-1*H*-benzotriazole (**HIIH**) and 4,7-dibromo-5,6-diiodo-1*H*-benzotriazole (**BIIB**). Both can be obtained from the commercially available 4,5-diiodobenzene-1,2-diamine^19^ under a single-step protocol, avoiding direct site-specific bromination of benzotriazole. Their binding affinities to hCK2α are comparable to that of TBBt, but **HIIH** displayed better physicochemical properties, being less hydrophobic, less acidic and much more soluble in aqueous medium^20^. Herein, we have used a wider set of physicochemical methods (Microscale Thermophoresis, MST, and Isothermal Titration Calorimetry, ITC) for the analysis of the thermodynamics of protein–ligand interactions. We have additionally determined the IC_50_ values against hCK2α for **HIIH** and **BIIB** and two non-iodinated reference ligands (**HBBH**, TBBt). Since TBBt decreases the viability^21^ and is often used as the reference kinase CK2 inhibitor^27–29^, we have tested cytotoxicity of all four compounds against four reference cancer cell lines. TBBt is classified in CTD database^22^ as the toxic compound that affects ATP/ADP ratio in cells^23^ and impairs neuronal excitability by increasing action potential threshold and lowering firing frequency^24^. Since its cytotoxicity can be explained not only by the suppression of activation of apoptotic proteins, but also by the decrease of mitochondrial membrane potential^25^, we additionally monitored how our compounds affect the mitochondrial inner membrane potential, which may be further related to their toxicity, especially in context of cardiotoxicity^26^.

## Results and discussion

**Figure 1:**
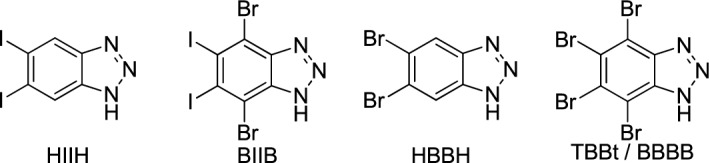
Structures of the studied compounds.

Four compounds, namely 5,6-diiodo-1*H*-benzotriazole (**HIIH)**, 4,7-dibromo-5,6-diiodo-1*H*-benzotriazole (**BIIB**), 5,6-dibromo-1*H*-benzotriazole (**HBBH)** and 4,5,6,7-tetrabromo-1*H*-benzotriazole (TBBt**, BBBB**) were synthesized according to the previously described protocols^20^. Structures of these four compounds are shown in Fig. 1. It is worth to mention that, since 4,5-diiodobenzene-1,2-diamine is commercially available^19^, the synthesis protocol of **HIIH** is reduced to the only last reaction step: the closure of the triazole ring performed with NaNO_2_ in AcOH/H_2_O at room temperature.

We have determined interactions between these four compounds and the catalytic subunit of human protein kinase CK2 using two different biophysical techniques: MST and ITC. MST, which enables direct estimation of binding affinity, K_d_, in a pseudo-titration mode has already been applied in detailed thermodynamic studies of the interactions between kinase hCK2α and its ligands^12,17^. The determined binding affinities are summarized in Table 1, and the pseudo-titration data are shown in Supplementary Figures S5-S8. For all ligands, contrary to the standard dose–response curve expected for a single binding site, a distinct “hill-like” shape was observed, indicating the presence of at least two different types of binding sites^17^. The first transition (small “up” effect reflecting binding in a solvent-protected pocket) corresponds to binding at the ATP-binding site, while the second (large “down” effect of binding on the protein surface) should be assigned to the solvent exposed region located at the interface between alpha and beta subunits of CK2. Both sites have recently been identified in crystal structures of variously brominated benzotriazoles bound by hCK2α^32^.

**Table 1:** Thermodynamic parameters for halogenated benzotriazoles in solution (in silico LogP), for their binding to the catalytic subunit of human protein kinase CK2 and viability of selected human cell lines, IC_50_ values, in response to tested compounds.

Parameter	Ligand				
	**HBBH**	TBBt	**HIIH**	**BIIB**	**cisPt**
LogP^a^^18^	2.88	4.56	3.93	5.59	
**DSF**^**a**^					
ΔT_m_ [K]	7.4 ± 0.1^18^	9.0 ± 0.1^18^	8.3 ± 0.1^20^	10.8 ± 0.1^20^	
**MST**					
K_d1_ (nM)	29 ± 13	16 ± 18	15 ± 7	3 ± 6	
K_d2_^b^ (nM)	327 ± 95	130 ± 88	374 ± 83	43 ± 22	
**ITC**					
K_d1_ (nM)	34 ± 4	16^c^	22 ± 4	1.3 ± 1.4	
ΔH1 (kJ/mol)	− 58 ± 1	− 22 ± 4	− 55 ± 1	− 14.0 ± 0.5	
ΔS1 (J/mol/K)	− 52 ± 4	75 ± 14	− 37 ± 4	123 ± 10	
K_d2_^b^ (nM)	–	93 ± 40	–	82 ± 42	
ΔH2 (kJ/mol)	–	− 4.4 ± 4.4	–	− 12.8 ± 0.6	
ΔS2 (J/mol/K)	–	118 ± 19	–	93 ± 5	
**In vitro inhibition of hCK2α**					
IC_50_(μM)	1.19 ± 0.53	0.49 ± 0.33	0.78 ± 0.29	0.23 ± 0.15	
Cell line	Cell viability data, IC50(μM)				
A-431	12 ± 2	7.8 ± 1.2	5.3 ± 0.7	2.2 ± 0.3	2.02 ± 0.06
HEPG2	18 ± 4	13 ± 2	3.3 ± 0.4	1.5 ± 0.3	2.43 ± 0.14
HCT116	19 ± 6	23 ± 9	6.1 ± 1.3	1.8 ± 0.3	4.7 ± 0.6
HCT116p53-/-	13 ± 3	48 ± 18	9.4 ± 1.4	2.5 ± 0.2	2.94 ± 0.13

^a^Literature data^18,20^.

^b^Two independent binding sites were identified, the second one should be assigned to the solvent exposed region located at the interface between alpha and beta subunits of CK2^32^.

^c^This value was constrained to the MST-derived K_d1_.

**Figure 2:**
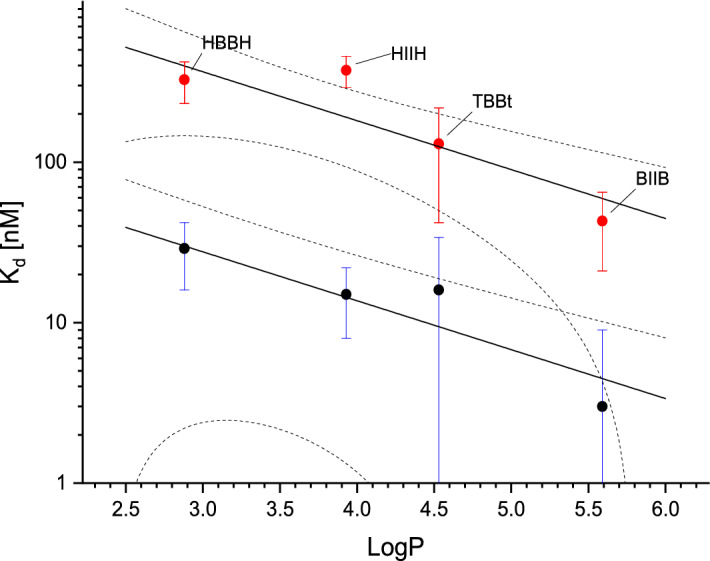
Correlation between calculated in silico ligand hydrophobicity and its binding by hCK2α; black points, K_d1,-_ the ATP-binding site, red points, K_d2_,—week binding on the surface.

Tetrahalogeno derivatives bind to hCK2α stronger than their dihalogeno analogues. For diiodo derivatives these differences, according to Student’s t-test, are statistically significant (K_d1_ = 15 ± 7 vs. 3 ± 6 nM, t(6,6) = 3.19, p = 0.01; K_d2_ = 374 ± 83 vs. 43 ± 22 nM, t(6,6) = 9.44, p < 0.001), while for bromo derivatives only affinity to weakly binding site differ significantly (K_d1_ = 29 ± 13 vs. 16 ± 18 nM, t(4,4) = 1.17, p = 0.29; K_d2_ = 327 ± 95 vs. 130 ± 88 nM, t(4,4) = 3.04, p = 0.02). Both diiodo derivatives bind to hCK2α stronger than their bromo counter partners (Fig. 2), however for the strongly binding site (affinity of which is close to the detection limit of our Monolith NT.115 hardware) these differences are not significant (t(4,6) = 2.24, p = 0.06 and t(4,6) = 1.68, p = 0.13 for di- and tetra-halogeno derivatives, respectively).

It is worth pointing that K_d2_ values determined for TBBt and **HBBH** moderately differ from those determined previously for the same system^17^. These discrepancies must be attributed to the different method of protein labelling with the fluorescent dye, of which that targeting lysine side-chains may compete with the ligand for a surface-exposed hydrophobic weak binding site. Thus, the application of amine-reactive RED-NHS dye (MO-L011) that targets surface-exposed lysine residues may cause modification of the residue(s) in the vicinity of K44, K71 (Fig. S18), and the bulky dye may compete with the ligand for this site. K78, which is crucial for ligand binding at the ATP-binding site, is more shielded from the modification. No such perturbations are expected for the currently used His-Tag Labeling Kit RED-tris-NTA 2nd Generation, which modifies solely the peripheral region of hCK2α.

**Figure 3:**
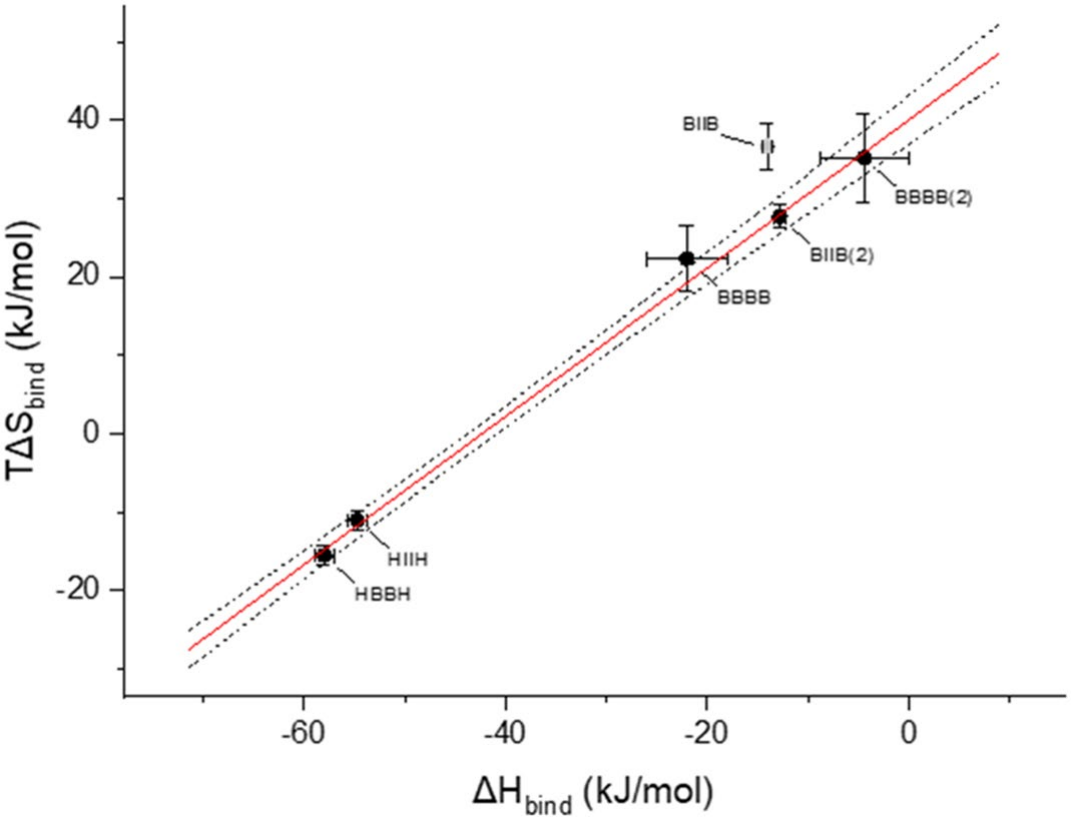
Entropy-enthalpy relation determined with Isothermal Titration Calorimetry for binding of halogenated benzotriazoles by hCK2α. Solid line identifies strong entropy-enthalpy compensation (slope 0.94) and chopped ones boarder its 95% confidence limits. Gray point (BIIB at the strongly binding site) was omitted from the analysis.

The binding of halogenated compounds was additionally followed with Isothermal Titration Calorimetry that enables determination of the stoichiometry, free energy and heat of ligand binding. We have successfully used this approach to study interactions of other ligands with hCK2α^12,17^. In line with MST data, two binding sites were identified for two the most hydrophobic tetrahalogenated compounds (TBBt and **BIIB**), which in MST experiments displayed the lowest K_d2_ values (i.e. the strongest binding). The model assuming a single binding site was found sufficient for the two remaining less hydrophobic derivatives, **HBBH** and **HIIH**. Inspection of the ITC-derived thermodynamic data, summarised in Table 1, shows for all but one ligand binging event the strong entropy-enthalpy compensation (slope 0.94 ± 0.04, Fig. 3). This common trend reaffirms our interpretation that the binding of halogenated benzotriazoles to the catalytic subunit of protein kinase CK2 is predominately driven by hydrophobic effect^16,18,20,30,31^. In this view, the excess heat of binding of **BIIB** at the ATP binding site (− 8 kJ/mol) may be assigned to the contribution of halogen bond(s), which are putatively formed between two iodine atoms of **BIIB** and two backbone carbonyl groups of residues located in the hinge region, resembling a virtual interaction that has been recently identified in the crystal structure of TBBt at hCK2α ATP binding site^32^.

**Figure 4:**
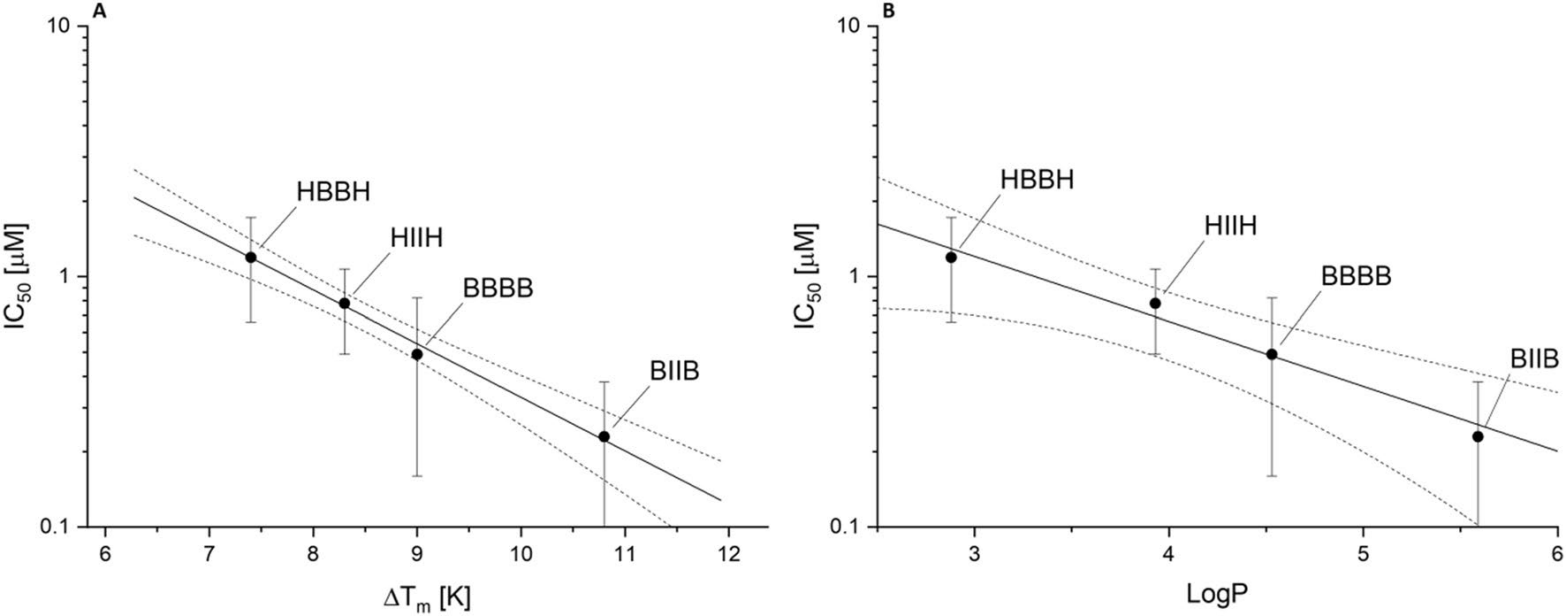
In vitro inhibition of hCK2a correlated with Thermal Shift Assay (**A**) and ligand hydrophobicity (**B**). Solid lines represent linear regression and chopped ones boarder their 95% confidence limits. Vertical lines display standard error of IC_50_ values.

We have additionally measured in vitro inhibition of hCK2α activity, IC_50_, by four studied compounds (Table 1, Suplementary Figures S9–S12). According to Student’s t-test no statistically significant differences between 5,6-diiodo- and 5,6,dibromo- derivatives were observed (t(5,5) = 1.52, p = 0.08 and t(4,5) = 1.59, p = 0.08 for di- and tertahalogeno benzotriazols, respectively), while 5,6-dihalogenated compounds differ significantly from their tetrahalogeno analogues (t(5,4) = 2.29, p = 0.03 and t(5,5) = 3.77, p = 0.003 for 5,6-dibromo- and 5,6-diiodo-benzotriazols, respectively). Overall, their inhibitory activity is moderately correlated with K_d1_ values, which results from the large bias of the determined K_d1_ values. However, IC_50_ is sufficiently correlated with the literature ΔT_m_ values^18,20^ determined with Thermal Shift Assay (nanoDSF) (Fig. 4A), which states the efficient method for screening of protein–ligand interactions. Moreover, IC_50_ values also correlate with calculated in silico LogP (Fig. 4B), pointing again that hydrophobic interactions drive binding of halogenated benzotriazoles.

**Figure 5:**
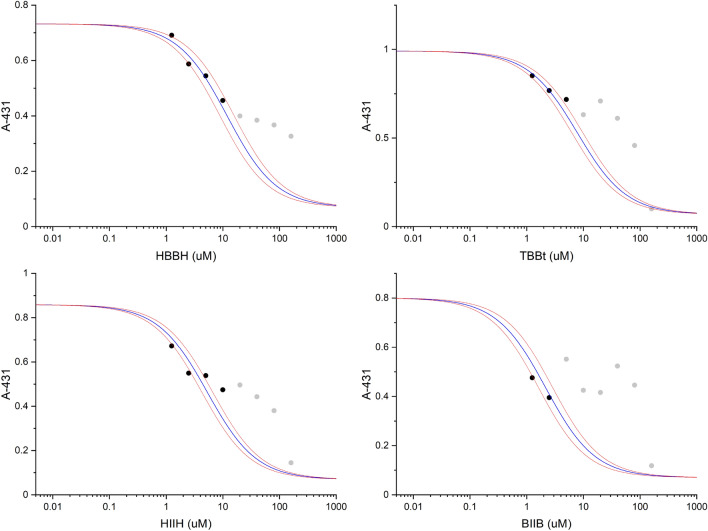
Viability of A-431 cell line in response to tested compounds (TBBt, HBBH, HIIH and BIIB) evaluated with MTT metabolism assay. Cells were incubated for 72 h with increasing concentrations (range = 1.25–160 μM) of compound. Black circles represents optical density at 570 nm, blue lines shows the standard dose–response curve fitted globally to three independent experiments (all experiments are shown in Figures S13–S16), and red lines boarder the confidence bands of the fitted line. Gray points denote removed high-concentration data, for which aggregation significantly perturbs resulting viability.

Since the biophysical methods confirmed that all tested ligands target hCK2α, we have further determined in vitro cytotoxic activity against four cancer cell lines: epidermoid carcinoma (A-431), hepatocellular carcinoma (HEPG2), and colorectal carcinoma (HCT116 and HCT116p53^–/–^). Additionally, the reference anticancer drug, cispaltin, was used as the positive control. Despite the fact that the obtained results were significantly biased due to a very limited aqueous solubility of the tested compounds, we were able to analyse the low-concentration part of the dose–response curves. Effect of aggregation of halogenated benzotriazole derivatives, which biased thermodynamic measurements at micromolar concentration of the ligand has been already described^17^. Aggregation of the studied compounds after dissolution in aqueous medium was monitored with DLS (Supplementary Figure S17). The shape of the autocorrelation curve is indicative for the existence of nano-aggregates. In all cases the shoulder at 100–600 µs is indicative of particles of the radius of 50–100 nm, however the similar ACF distribution was observed at different solute concentration: the highest for **HBBH** (160 µM), and the lowest for the most hydrophobic **BIIB** (40 µM). It should be pointed that aggregation makes ligand unavailable for cells, so further addition did not affected the viability, since the concentration of dissolved monomeric compound remains saturated. This effect was the most significant in case of the less soluble **BIIB**, however for any of tested compounds a complete dose–response effect undistorted in the high-concentration region could not be recorded. To overcome this limitation we have used the simplified approach, in which IC_50_ values were fitted globally for the restricted set of low-concentration data obtained in three independent experiments, while up and down asymptotes were constrained to OD values measured in each experiment for untreated cell culture and for pure buffer, respectively. The representative viability data, based on MTT assay, obtained for epidermoid carcinoma A-431 cell line are shown in Fig. 5, while all other results are presented in Supporting Figures S7–S10. The calculated IC_50_ values are summarized in Table 1.

While the brominated compounds (**HBBH** and TBBt) similarly reduce cell viability, the iodinated ones display higher cytotoxicity. This effect is correlated with their higher activity in the enzymatic assay, however for all tested cell lines **HIIH** reduces the viability more efficiently than TBBt, and **BIIB** reduces viability of all cell lines even more, with IC_50_ values estimated close to the values obtained for cisplatin, the reference anticancer drug. It is worth to mention, that the extremely low solubility of this compound preclude its application as a potential anticancer drug. Deletion of p53 in HCT116 did not significantly affected cell viability, so mechanism of inhibition of these compounds is probably not correlated with this protein.

**Figure 6:**
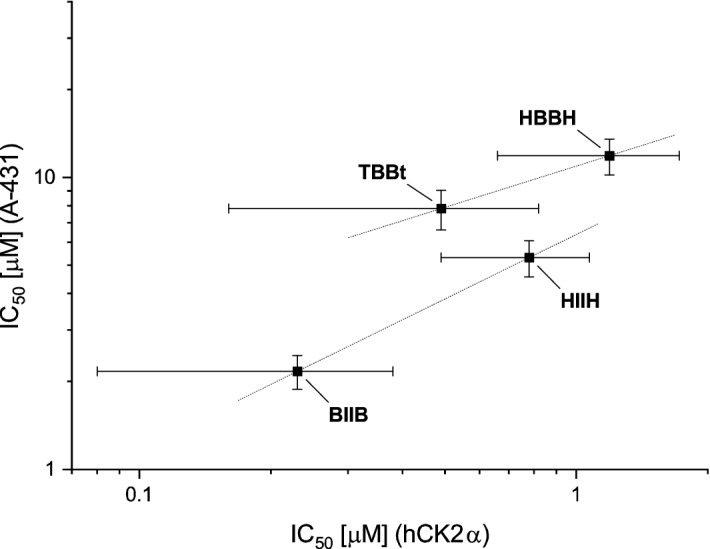
The correlation of IC_50_ values in the enzymatic assay against hCK2α and in the viability test for A-431 cancer cell line. The qualitative difference between non-iodinated and iodinated compounds is indicated by dotted lines. Experimental error is represented by horizontal and vertical lines.

According to Fisher F-test, no significant differences were observed between the activity against individual cell lines for **HBBH**, **HIIH** and **BIIB** (F(3,52) = 0.80, p = 0.50; F(3,54) = 2.68, p = 0.07 and F(3,32) = 0.96, p = 0.42, respectively), while the for TBBt IC_50_ values differ significantly (F(3,44) = 12.6, p < 10–4). However, for each cell line these four compounds differ in their cytotoxic activity (F(3,46) = 16.3, p < 10^–6^). Post-hoc analysis (t-test) shows that at significance level α of = 0.02 compounds’ activity can be ordered as **HBBH** < TBBt < **HIIH** < **BIIB** (t(3,3) = 3.10, 3.53 and 7.03 and p = 0.018, 0.012, 0.001, respectively). So, brominated compounds are less active than their diiodo counter partners. We have compared IC_50_ values determined in enzymatic (hCK2α inhibition) and viability (A-431 cancer cell lines) tests (Fig. 6). Interestingly, when compared with hCK2α inhibition, diiodo derivatives affect viability of cancer cells much more than the corresponding bromo-benzotriazoles. These qualitative differences indicate that effects other than direct CK2 inhibition affect significantly cytotoxicity of halogenated benzotriazoles, to which among other contribute permeability and side effect on ATP synthesis.

**Figure 7:**
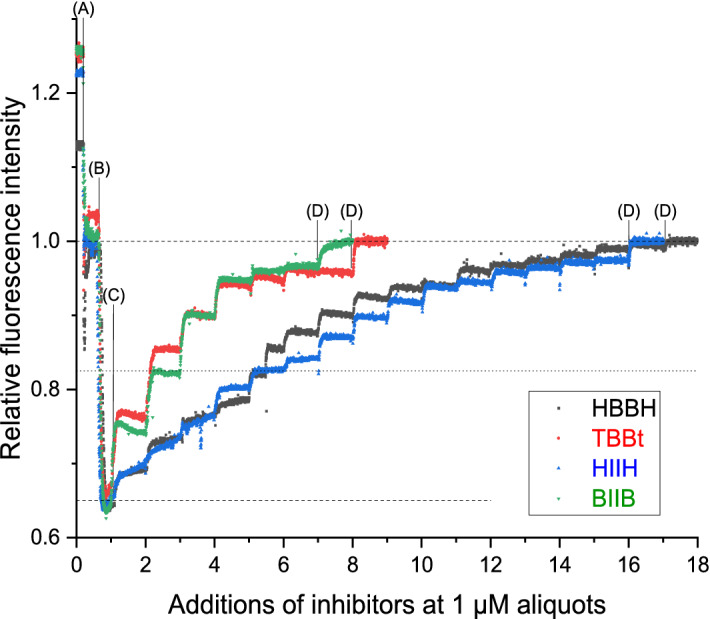
Variation of the mitochondrial inner membrane potential upon treatment with HBBH, TBBt, HIIH and BIIB. The additions were 0.5 mg/ml Rhodamine 123, 0.15 mg/ml mitochondrial proteins (**A**), 10 µl/ml of ethanol (**B**), the indicated inhibitors at 1 µM aliquots (**C**) (only first addition is marked in the chart) and 3 mM CCCP (**D**). Black points—HBBH, red points—TBBt, blue points—HIIH, green points—BIIB. Shown are representative of two/three independent experiments performed for each compound.

To evaluate a contribution to the toxicity of the tested compounds not directly connected with kinase inhibition, we have monitored how they decrease the mitochondrial inner membrane (IM) potential (ΔΨ). This was monitored by fluorescence quenching of Rhodamine 123 in mitochondria isolated from the yeast *Saccharomyces cerevisiae.* In response to variations in ΔΨ this dye accumulates proportionally inside the mitochondrial matrix, where its fluorescence is quenched, thus fluorescence changes reflect the changes in the IM potential^33,34^. The traces shown in the Fig. 7 demonstrate how the fluorescence of Rhodamine 123 varied upon the following additives: (A) mitochondria, (B) EtOH, which provided the substrates for respiratory chain and led to creation of membrane potential (ΔΨ) and hence concomitant fluorescence quenching, (C) the particular compound, (D) carbonyl cyanide-mchlorophenyl hydrazone (CCCP)—the standard membrane potential uncoupler leading to loose of ΔΨ and retaining of Rhodamine 123 fluorescence—for the normalisation of each experiment. All the data were normalized relative to the fluorescence intensity after addition CCCP (set to 1).

Addition of mitochondria (A) decreased the relative fluorescence of Rhodamine 123 to ~ 1.0 (dashed line). Energization of mitochondria by EtOH (B) lead to further decrease of fluorescence to ~ 0.65 (dashed line), due to polarisation of the inner membrane (IM) by respiratory chain and the consequent uptake of the dye inside the matrix. Addition of each compound (C) in 1 μl portions of 1 mM solution lead to the stepwise drop of the ΔΨ and consequent increase of the fluorescence. Each ligand displayed individual ability to uncouple ΔΨ. The stronger uncouplers are **BIIB** and TBBt, both of them are anionic tetrahalogenated benzotriazole derivatives. At c.a 4 µM concentration, addition of ligand only minutely affects ΔΨ, at the level ~ 0.95. Further addition of CCCP retains fluorescence to ~ 1 (dashed line, what was the value for the fully uncoupled membrane potential). **HIIH** and **HBBH** much weaker affect ΔΨ, a fluorescence c.a. 0.95 was reached at ligand concentration c.a. 12 µM and the slight effect of further addition was reached up to concentration of c.a. 15 µM, when the membrane potential is almost fully uncoupled (only minor effect of further CCCP addition). To quantify the effect of ligand addition we have compared the ligand concentrations required to recover 50% of the initial fluorescence drop (dotted line), i.e. the average of value after addition of EtOH and fully decoupled ΔΨ. In the case of tetrahalohenated benzotriazoles it is less than 2 µM, while for the dihalogenated ones is much higher (> 5 µM). So, the predominately neutral **HBBH** and **HIIH** are at least twice less effective in decoupling ΔΨ than the anionic **BIIB** and TBBt.

## Conclusions

Obtained results clearly indicate that **HIIH** can be successfully used as the leading compound for CK2 inhibitors, stating an attractive alternative for TBBt. Its interaction with hCK2α is similar to that of TBBt. Only slight differences in protein–ligand interaction may be caused by different physicochemical properties of **HIIH.** This includes the lower hydrophobicity, which is additional advantage of this compound. Moreover, synthesis of **HIIH** is much simpler than synthesis of TBBt based on bromination of benzotriazole. We showed that **HIIH** much more decreases viability of cancer cell lines than TBBt (p < 0.012). Moreover, its higher activity is not correlated with decoupling of mitochondrial inner membrane potential, since it decouples ΔΨ much less than TBBt does. So, its activity should be assigned directly to protein inhibition. Moreover, weaker impact on mitochondria makes **HBBH** less toxic than TBBt, the toxicity of which was associated with decrease in cellular ATP^25^. Even though we have identified **BIIB** (4,7-dibromo-5,6-diiodo-1*H*-benzotriazole) as much stronger CK2 inhibitor, which also very efficiently decreases cancer cells viability, and decouples ΔΨ not more than TBBt does. However, its synthesis is more demanding, basing on bromination of **HIIH**. **BIIB** is also more hydrophobic and less soluble than **HIIH** and TBBt, what makes it less useful as a leading compound for CK2 inhibitors. In general, the replacement of bromine atom with iodine (at least in the halogenated benzotriazoles) increases cytotoxic activity much more than it could be expected from the inhibitory activity determined in the enzymatic assay. This points the synergistic effect of the iodine atom which improves both the inhibitory activity of the compound and its transfer through the cell membrane.

## Methods

### Chemistry

All starting materials and solvents for reactions were purchased from Sigma Aldrich, Fluorochem, ABCR or Chempur. All compounds were synthesized according to protocols previously described by our team^20,30^. Structure of compounds was confirmed using ^1^H NMR spectroscopy and Mass spectrometry. ^1^H NMR spectra were recorded with Varian 500 MHz spectrometer. Either TMS or the residual solvent signal were used as the internal standard. High resolution mass spectra (HR:MS) were recorded on an TQ OrbitrapVelos instrument (Thermo Scientific). Purity of compounds was confirmed by HPLC, see Supporting Information for details.

### Protein sample preparation

The catalytic subunit of human protein kinase His6-tagged at the C-termini CK2 was expressed and purified according to the method described previously^16^. Plasmid pET28 containing DNA encoding human CK2α was transformed into BL21(DE3) *Escherichia coli* cells. A single colony was inoculated in 30 ml LB broth supplemented with kanamycin (50 μg/ml) and incubated overnight at 37 °C. The culture (5 ml) was used to inoculate 1000 ml of Luria Broth with kanamycin (50 μg/ml) and cells were cultured at 37 °C until OD_600_ reached 0.5, when hCK2α expression was induced by 0.5 mM isopropylthio-β-galactoside. Cells were then incubated at 37 °C for 4 h, harvested by centrifugation at 6000 rpm for 10 min at 4 °C, and stored at -20 °C.

Thawed cells were suspended in 30 ml of buffer A (25 mM Tris–HCl pH 8, 500 mM NaCl, 10 mM imidazole, 5 mM β-mercaptoethanol) with 0.2 mM PMSF (phenylmethylsulfonyl fluoride) and protease inhibitor cocktail (Sigma-Aldrich P8849), and sonicated by five 1-min bursts. The cell lysate was centrifuged at 20,000 rpm for 40 min, and the supernatant fraction processed by Ni^2+^-NTA agarose chromatography under non-denaturing conditions. The protein was then purified by affinity chromatography with a HiTrap Heparin HP column (GE HealthCare), and eluted with a linear 0.4 to 1 M NaCl gradient buffered with 25 mM Tris–HCl, pH 8.5. Purified (His)6-tagged hCK2α was concentrated by ultrafiltration, rebuffered in 500 mM NaCl, 50% glycerol, 1 mM DTT, 25 mM Tris–HCl, pH 8.5, and stored at − 80 °C. The final yield of purified protein was 20 mg/l. Concentration of the protein was estimated by UV absorbance at 280 nm, assuming molar extinction coefficient ε = 61,895 M^−1^ cm^−1^.

### Microscale thermophoresis (MST)

Samples of protein (50 nM in 25 mM Hepes buffer pH 8 with 0.5 M NaCl and 1%DMSO content v/v) labeled with Monolith His-Tag Labeling Kit RED-tris-NTA kit (NanoTemper Technologies) were prepared with varying ligand concentration according to previously described protocol^16^. The measurements were performed using Monolith NT.115 (NanoTemper Technologies) apparatus in standard capillaries (Nanotemper Technologies). For each ligand six independent pseudo-titration experiments, each with 16 ligand dilutions, were performed. The thermophoretic effect was then analyzed globally assuming the common values of binding affinities for two independent sites (K_d1_, K_d2_), while the asymptotic values for the *apo* form of the protein and 1:1 and 1:2 complexes with the ligand were estimated individually for each experiment. The model of two independent binding sites, implemented in origin, has been already described and tested^17^. It is worth noting that the used method of data treatment goes beyond the standard MST software capabilities.

### Isothermal titration calorimetry (ITC)

All ITC measurements were carried out in 25 mM Tris buffer (pH 8, 0.5 M NaCl, 1% DMSO concentration) at 25 °C with the MicroCal iTC200 (Malvern) according to the reverse mode of ITC experiment^17,32^, in which ligand solution (6–16 µM) placed in the sample cell was titrated with the protein (50–150 µM) – both samples were dissolved in the same buffer to avoid undesired dilution effects. For each ligand five independent titration experiments were done. The raw thermograms were preprocessed with MicroCal ITC-ORIGIN, and the thermodynamic parameters were then estimated globally using previously reported procedure^32^. The model of single binding site was applied for **HIIH** and **HBBH**, while for **BIIB** and TBBt the model of two independent binding sites was scored better according to F-test, however for TBBt, due to small heat of binding, the affinity of the strongly binding site have to be constrained at the value determined in MST experiment.

### Dynamic light scattering (DLS)

Experiments were carried out at 25˚C with the DynaPro NanoStar 192-DPN apparatus (Wyatt Technology) equipped with 661 nm laser. The autocorrelation function for the light scattered by solute placed in Eppendorf disposable cuvettes (50–2000 μl) was measured in the range of 0.5 μs– 0.1 s at solute concentration for which short-time ACF signal approached 0.2. Solutions with 2% (v/v) DMSO content were prepared in 25 mM Tris–HCl (pH 8, 0.5 M NaCl) filtered with 220 nm pore size syringe filter. All samples were centrifuged (9000 g) for 3 min immediately before the experiment.

### In vitro IC_50_ assay

The compound inhibitory activity was monitored based on the luminescence measured with the SpectraMax iD3 Multi-Mode Microplate Reader (Molecular Devices, San Jose, CA, USA), using the previously described method with the aid of ADP-Glo kinase assay (Promega, Walldorf, Germany)^35^. The measurements were carried in 96-well plate in volume of 25 μl in 20 mM Tris–HCl buffer (pH 7.5), containing 100 ng of hCK2α (1 μl), 10 μM CK2 substrate peptide RRRDDDSDDD (Biaffin GmbH & Co KG), 10 μM ATP, 20 mM MgCl_2_ and 2.5 μl of ligand serially diluted in DMSO. The reaction was initiated by adding the enzyme and kept going for 20 min at 30 °C. For each ligand the standard dose–response curve was globally fitted to at least four independent experiments: common IC_50_ value was optimized for all experiments, while top and bottom luminescence asymptotes were fitted individually for each experiment.

### Cell viability assay

The viability of cells was assayed by measuring the conversion of MTT (3‐(4,5‐dimethylthiazol‐2‐yl)‐2,5‐diphenyltetrazolium bromide) to the formazan (the rate of this reaction is proportional to the number of surviving cells). Cells were seeded in a 96-well plate at a density of 2500–3500 cells per well, 24 h before treatment. Treatment with measured compounds and cisplatin (2.5–160 µM) was performed for 72 h. MTT stock solution (Sigma-Aldrich) was added at the final concentration 0.5 mg/mL. After 4 h of incubation at 37 °C, water‐insoluble formazan was dissolved in a lysis buffer containing 20% SDS, 50% DMF, 2.5% hydrochloric acid and 2.5% acetic acid. Optical densities were measured at 570 nm using a scanning multi‐well spectrophotometer (PARADIGM Detection Platform; Beckman Coulter, Brea, CA). For each ligand the standard dose–response curve was fitted to three independent experiments, however the concentration range was restricted to overcome the effect of uncontrolled aggregation. **HBBH** and **HIIH** were analysed in the concentration range of 1–10 µM, while **TBBt** and **BIIB** in the range of 1–5 and 1–2.5 µM, respectively. We have applied the simplified approach to estimate inhibitory activity: top and bottom asymptotes were constrained to the individual OD values measured for untreated cells and pure buffer, respectively, while the common IC_50_ value was for each compound optimized globally for three independent experiments.

### Isolation of mitochondria from yeast cells and measurement of mitochondrial inner membrane potential

The wild type strain MR6 (*MATa* ade2-1 his3-11,15 trp1-1 leu2-3,112 ura3-1 CAN1 arg8::HIS3^36^ was grown in YPGalA (1% Bacto yeast extract, 1% Bacto Peptone, 2% galactose, 40 mg/L adenine) liquid medium to OD_600_ = 4 at 28 °C. The mitochondria were prepared from yeast cells by the enzymatic method described in reference^37^. Variations in transmembrane potential (ΔΨ) were evaluated in the quartz cuvette containing 2 ml of respiration buffer (10 mM Tris-maleate (pH 6.8), 0.65 M sorbitol, 0.3 mM EGTA, and 3 mM potassium phosphate), Rhodamine 123 (0.5 μg/ml) and 0.150 mg/ml of mitochondria energized by addition of ethanol (EtOH, 10 μl/ml). At the end of each experiment the carbonyl cyanide-m-chlorophenyl hydrazone (CCCP, the IM potential uncoupler) was added at 4 μM for the normalization. The Rhodamine 123 fluorescence was read at λexc of 485 nm and λem of 533 nm under constant stirring at stable temperature of 28 °C using a Cary Eclipse Fluorescence Spectrophotometer (Agilent Technologies, Santa Clara, CA, USA)^38^.

## Supplementary Information

Supplementary Information.
