# Supplementary material for: 5,6-diiodo-1H-benzotriazole: new TBBt analogue that minutely affects mitochondrial activity
Source: Sci Rep. 2021 Dec 8;11:23701. doi: 10.1038/s41598-021-03136-8 (PMC8654832; doi:10.1038/s41598-021-03136-8)
Supplement: Supplementary file 1 — Supplementary Information. [file 41598_2021_3136_MOESM1_ESM.docx]

Supporting Information for

5,6-diiodo-1*H*-benzotriazole – new TBBt analogue that minutely affects mitochondrial activity

Daniel Paprocki, Maria Winiewska-Szajewska, Elżbieta Speina, Róża Kucharczyk, Jarosław Poznański

Institute of Biochemistry and Biophysics, Polish Academy of Sciences, Pawinskiego 5a, 02-106 Warsaw, Poland; email: jarek@ibb.waw.pl.

Table of contents

[1. Synthesis – Schematic Overview 2](#_Toc86146526)

[2. HPLC Traces of all Synthesized Compounds 3](#_Toc86146527)

[3. MST data 5](#_Toc86146528)

[4. Enzymatic *in vitro* activity assay 9](#_Toc86146529)

[5. Cell viability 11](#_Toc86146530)

[6. DLS data 15](#_Toc86146531)

[7. Crystal structure of hCK2α and TBBt 16](#_Toc86146532)

# Synthesis – Schematic Overview

**Scheme S1**. Synthesis of all measured compounds. a) 1,65 eq. NaNO_2_, H_2_O, AcOH, RT b) 1.5 eq./substitued atom Br_2_, conc. HNO_3_.80 °C.

# HPLC Traces of all Synthesized Compounds

**Fig. S1**. HPLC trace of compound HBBH. Wavelength 280 nm – black line, wavelength 230 nm – red line.

**Fig. S2**. HPLC trace of TBBt. Wavelength 280 nm – black line, wavelength 230 nm – red line.

**Fig. S3**. HPLC trace of compound HIIH. Wavelength 280 nm.

**Fig. S4**. HPLC trace of compound BIIB. Wavelength 280 nm.

# MST data

**

**

**Fig. S5**. MST pseudo-titration data collected for **HBBH**. Black circles shows experimental data, grey ones indicate data removed from the analysis, thick line represents the fitted model of two independent biding sites and dotted ones boarder the 95% confidence limits for the model.


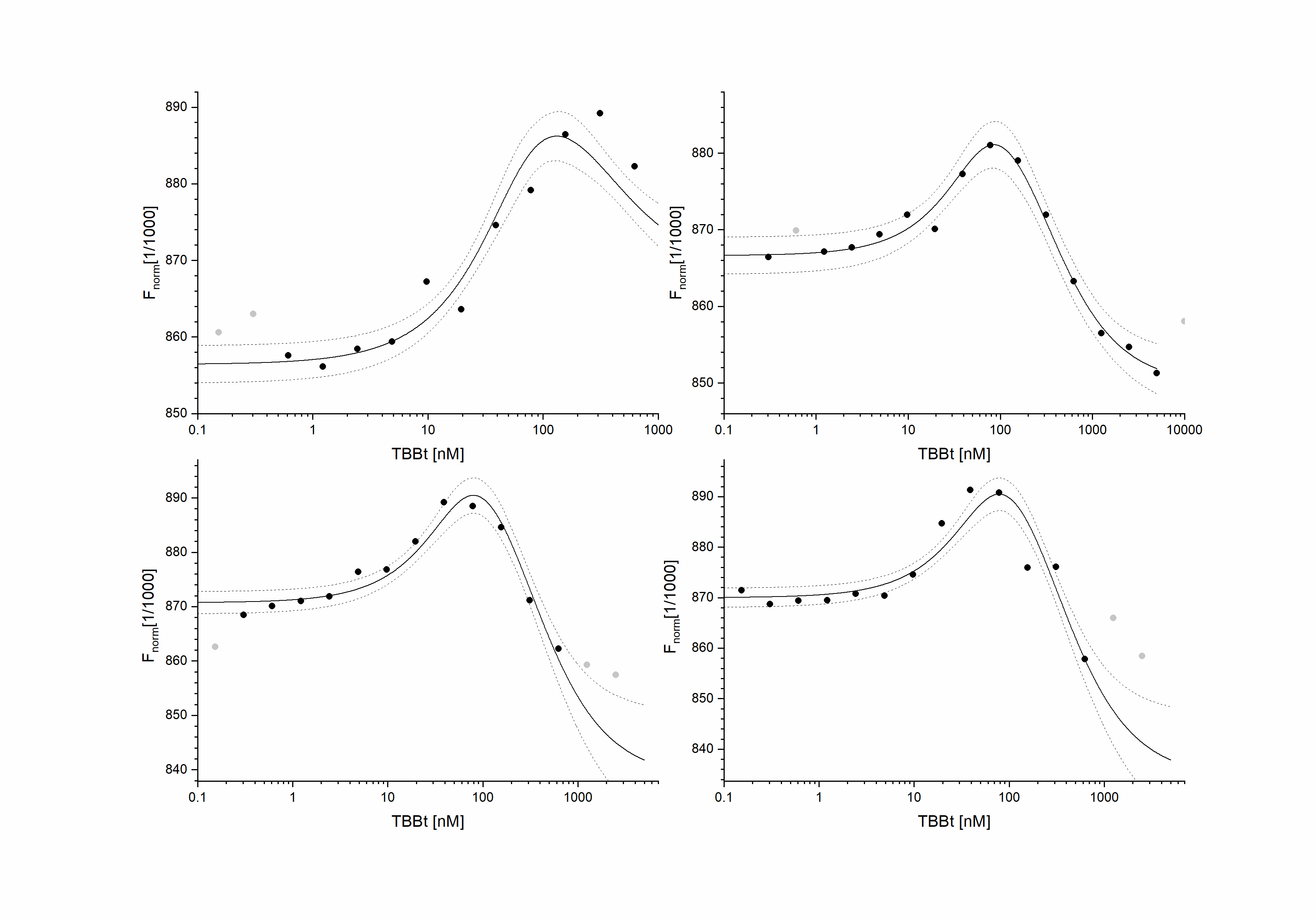


**Fig. S6**. MST pseudo-titration data collected for TBBt. Black circles shows experimental data, grey ones indicate data removed from the analysis, thick line represents the fitted model of two independent biding sites and dotted ones boarder the 95% confidence limits for the model





**Fig. S7**. MST pseudo-titration data collected for **HIIH**. Black circles shows experimental data, grey ones indicate data removed from the analysis, thick line represents the fitted model of two independent biding sites and dotted ones boarder the 95% confidence limits for the model


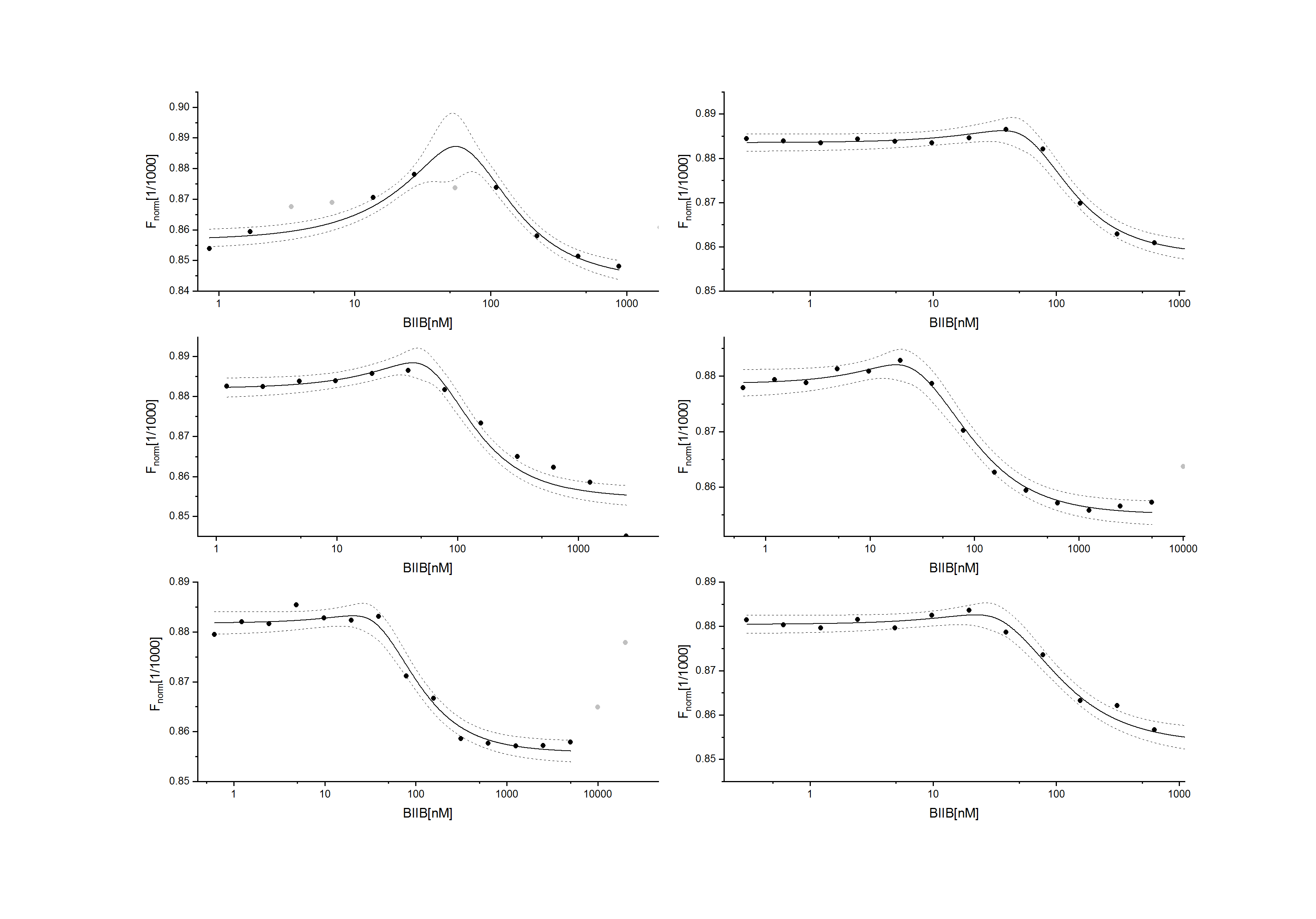


**Fig. S8**. MST pseudo-titration data collected for **BIIB**. Black circles shows experimental data, grey ones indicate data removed from the analysis, thick lines represent the fitted model of two independent biding sites and dotted ones boarder the 95% confidence limits for the model

# Enzymatic *in vitro* activity assay

**

**Figure S9 The inhibition of hCK2α by compound **HBBH**. Black circles shows experimental data, thick lines represent the globally fitted model and dotted ones boarder the 95% confidence limits for the model.





Figure S10 The inhibition of hCK2α by compound **BBBB**. Black circles shows experimental data, thick lines represent the globally fitted model and dotted ones boarder the 95% confidence limits for the model.





Figure S11 The inhibition of hCK2α by compound **HIIH**. Black circles shows experimental data, thick lines represent the globally fitted model and dotted ones boarder the 95% confidence limits for the model.





Figure S12 The inhibition of hCK2α by compound **BIIB**. Black circles shows experimental data, thick lines represent the globally fitted model and dotted ones boarder the 95% confidence limits for the model.

# Cell viability





Figure S13 Viability of A-431 cell line in the presence of four tested compounds (HBBH, TBBt, HIIH, BIIB) and cisplatin**.** Each row shows three independent experiments), solid circles denotes experimental data, solid lines follow the model fitted globally to six experiments, while dashed lines board 95% confidence bands for the model.





Figure S14 Viability of HEPG2 cell line in the presence of four tested compounds (HBBH, TBBt, HIIH, BIIB) and cisplatin**.** Each row shows three independent experiments), solid circles denotes experimental data, solid lines follow the model fitted globally to six experiments, while dashed lines board 95% confidence bands for the model.





Figure S15 Viability of HTC116 cell line in the presence of four tested compounds (HBBH, TBBt, HIIH, BIIB) and cisplatin**.** Each row shows three independent experiments), solid circles denotes experimental data, solid lines follow the model fitted globally to six experiments, while dashed lines board 95% confidence bands for the model.





Figure S16 Viability of HTC116p53^-/-^ cell line in the presence of four tested compounds (HBBH, TBBt, HIIH, BIIB) and cisplatin**.** Each row shows three independent experiments), solid circles denotes experimental data, solid lines follow the model fitted globally to six experiments, while dashed lines board 95% confidence bands for the model.

# DLS data

**Figure S17**. DLS-derived autocorrelation curves measured for the studied compounds (blue) and pure buffer (black). The shoulders observed in the range of 100-500 µs indicate the existence of nanoaggregates of a radius of 50-100 nm. Any solutes of this size cannot be observed in pure buffer.

# Crystal structure of hCK2α and TBBt


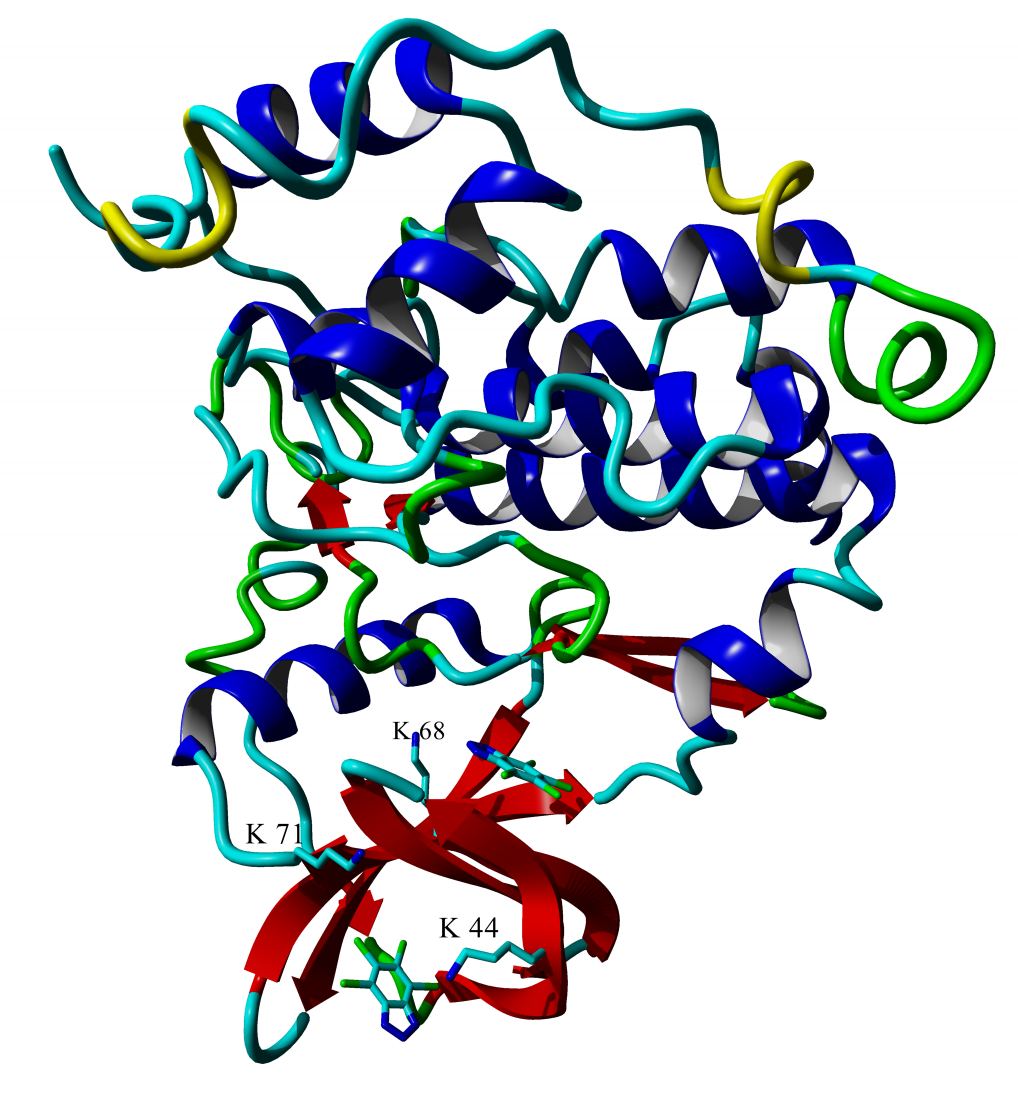


**Figure S18**. Two binding sites for TBBt identified in crystal structure of hCK2α (pdb 6tll). Modification of two surface-exposed lysine residues (K44 and K71) with hydrophobic large fluorescent dye may interfere with the ligand binding on the protein surface. K68, located in the ATP-binding cavity, is protected from such modification.
